# Extracellular Vesicles in chondrogenesis and Cartilage regeneration

**DOI:** 10.1111/jcmm.16290

**Published:** 2021-05-04

**Authors:** Hong Song, Jiasong Zhao, Jun Cheng, Zhijie Feng, Jianhua Wang, Amir Abbas Momtazi‐Borojeni, Yimin Liang

**Affiliations:** ^1^ Department of Orthopedics Guizhou Province Orthopedics Hospital Guiyang, Guizhou China; ^2^ Department of International Ward Hospital of Chengdu University of Traditional Chinese Medicine Chengdu China; ^3^ Department of Spine Surgery Chongqing Three Gorges Central Hospital Chongqing China; ^4^ Department of Geriatric Orthopaedics Tangshan City Second Hospital Hebei Province Tangshan China; ^5^ Department Bone Microsurgery Sanya people's Hospital Sanya China; ^6^ Department of Medical Biotechnology School of Medicine Mashhad University of Medical Sciences Mashhad Iran; ^7^ Department of Orthopedics Huangyan Hospital of Wenzhou Medical University Taizhou First People’s Hospital Taizhou China

**Keywords:** Cartilage regeneration, Chondrogenesis, Exosome, Extracellular vesicles, Regenerative medicine

## Abstract

Extracellular vesicles (EVs), mainly exosomes and microvesicles, are bilayer lipids containing biologically active information, including nucleic acids and proteins. They are involved in cell communication and signalling, mediating many biological functions including cell growth, migration and proliferation. Recently, EVs have received great attention in the field of tissue engineering and regenerative medicine. Many in vivo and in vitro studies have attempted to evaluate the chondrogenesis potential of these microstructures and their roles in cartilage regeneration. EVs derived from mesenchymal stem cells (MSCs) or chondrocytes have been found to induce chondrocyte proliferation and chondrogenic differentiation of stem cells in vitro. Preclinical studies have shown that exosomes derived from MSCs have promising results in cartilage repair and in cell‐free therapy of osteoarthritis. This review will focus on the in vitro and in vivo chondrogenesis and cartilage regeneration of EVs as well as their potential in the treatment of osteoarthritis.

## 1 INTRODUCTION

Cartilage is a hydrated tissue with no vascular and neural networks. They are divided into three major groups: hyaline, fibrous and elastic cartilages. Hyaline cartilage is the most frequent form and is found in synovial joints, ribs, nose, trachea, bronchi, etc^1^ The main roles of this articular hyaline cartilage are to tolerate bone load and forming a lubricant environment to enable joint movement.^2^ Extracellular matrix (ECM) synthesized by chondrocytes constitutes the main part of each cartilage. Collagen type II is the most frequent ECM molecule in hyaline cartilage and accounts for 90%‐95% of total collagen molecules.^3^, ^4^ Collagen II forms filamentous structures with collagen IX, responsible for cartilage tensile and shear stress. Proteoglycans, such as aggrecan, and glycosaminoglycans (GAG), such as chondroitin sulphate, are the other components of the articular cartilage ECM.^5^, ^6^ The integrity of ECM is vital for the normal function of cartilage. Therefore, changes in ECM elements and composition are the main feature of cartilage diseases.^7^

Osteoarthritis (OA) is characterized by progressive cartilage damage and is the most prevalent cartilage‐related disease. OA mainly affects elderly people and is more prevalent in women than men.^8^ Trauma and pathological conditions such as obesity and congenital abnormalities are the main cause of the disease and usually, knee and hip cartilages are affected.^9^ Chondrocytes that produce and secrete ECMs are the main cell source of cartilage; however, they constitute only 2% of cartilage volume. They need a suitable microenvironment for their survival and function. This microenvironment is changed during cartilage diseases results in chondrocyte apoptosis. On the other hand, they have limited replication capacity.^10^, ^11^ In addition, inflammation which is one of the main characteristics of OA causes more changes in cartilage ECM and therefore exacerbates tissue damage.^12^ Despite the high prevalence of cartilage diseases such as OA, no definitive therapy is available. Surgical and non‐surgical therapies are associated with side effects, and their efficacy is not satisfactory.^13^ As many cartilage diseases are associated with chondrocyte loss or dysfunction, cell‐based therapy has been suggested as an alternative therapy. Stem cells including embryonic stem cells (ESCs), induced pluripotent stem (iPS) cells and mesenchymal stem cells (MSCs) are widely used in tissue engineering and regenerative medicine to restore and repair injured tissues.^14^ These cells are mainly used to differentiate into somatic cells of the organ. Furthermore, some types of stem cells, such as MSCs, can induce endogenous progenitors and stem cells to migrate, proliferate and differentiate.^15^ Since many functions of MSCs are mediated through paracrine effects and with regard to cell transplantation complications such as immune rejection, cell‐free secretome has been proposed for the treatment of cartilage diseases.^16^

## 2 THE BIOLOGY AND PATHOPHYSIOLOGY OF EXTRACELLULAR VESICLES IN OA

The secretome includes extracellular vesicles (EVs) and is secreted from many cell types.^17^ EVs are considered as small bilayer lipids with 30‐1000 nm diameters. EVs are usually defined as exosomes, microvesicles and apoptotic bodies.^18^ Among them, exosomes and microvesicles share many characteristics, but vary in size as well as protein composition. Exosomes are 30‐100 nm cup‐shaped vesicles, while microvesicles are heterogeneous with 50‐1000 nm in diameter.^19^ Exosomes are released from their origin cells by fusion with the plasma membranes, while microvesicles are released through shedding from the plasma membrane. Coding and non‐coding RNAs, proteins, antigen‐presenting molecules and DNA are the main compositions of exosomes.^20^, ^21^, ^22^ Microvesicles consist of bilayer lipids containing mRNAs, miRNAs as well as lipids and cytosol. Because of their size and composition, exosomes are considered to be more important in the field of tissue engineering and regenerative medicine. Almost all of the cells, both in normal and pathological states, including lymphocytes, antigen‐presenting cells, platelets, mesenchymal stem cells (MSCs), many mature somatic cells and tumour cells, release exosomes and microvesicles into almost all body fluids. The cargo of each exosome reflects its origin cells. They include cell‐specific receptors, heat shock proteins (HSPs), tetraspanins (CD markers), lipid rafts such as flotillin‐1 and integrins, which mediate exosomes‐cell interactions in a paracrine manner.^19^, ^23^, ^24^

Exosomes are present in the synovial fluid (SF); however, the quantity and compositions of SF‐exosomes and more importantly their functions are changed in cartilage‐related diseases. Studies have shown a higher levels of exosomes in patients with early and late‐stage OA than in the normal population.^25^ Changes in proteins, miRNAs and lnRNAs have been also observed in the SF of patients with joint diseases.^25^, ^26^ In addition, the composition of exosomes differs between various joint disease.^27^ In patients with joint diseases, exosomes derived from fibroblast‐like synoviocytes (FLS) activate CD4^+^ lymphocytes and increase the secretion of inflammatory cytokines.^28^ Furthermore, these exosomes mediate bone and cartilage degradation through inducing matrix metalloproteinases and promoting the osteoclast function, respectively.^29^ Plasma‐derived exosomes in patients with RA increase the activity of pro‐inflammatory cytokines produced by peripheral blood immune cells.^30^ In addition, these exosomes can activate complement systems and increase infiltration of immune cells such as neutrophils and M1 macrophages, which further degrade cartilage.^31^ These data suggest the role of exosomes in the joint disease pathology and provide a perspective on the treatment of affected patients.

Recently, many studies have evaluated the potential use of exosomes as diagnostic markers as well as carriers of genes for the therapeutic purpose.^32^, ^33^, ^34^ It is also used to suppress immune responses during cell and organ transplantation to avoid immune rejection.^35^, ^36^ Exosomes could be used to regenerate and repair tissues including bone and cartilage.^37^, ^38^ As exosomes play key roles in the modulation of inflammation and immune responses, they have been widely used by researchers in the treatment of autoimmune and inflammatory diseases such as OA.^39^, ^40^ In the following sections, the in vitro and in vivo potential roles of exosomes in chondrogenesis and healing OA are discussed.

## 3 THE IN VITRO CHONDROGENIC POTENTIAL OF EVS

In the field of cartilage tissue engineering, iPS and various sources of MSCs have been widely used to differentiate into mature chondrocytes.^41^, ^42^, ^43^ Some studies have used growth factors and miRNAs to induce chondrogenic differentiation.^43^, ^44^, ^45^ Some in vitro studies have shown that EVs or exosomal miRNAs can enhance chondrogenesis and chondrogenic differentiation directly or indirectly (Table 1). Huilei Yu *et al* showed that MSC‐derived exosomes promote proliferation and chondrogenic differentiation of tendon stem/progenitor cells (TSPCs) into mature chondrocytes.^46^ Cosenza *et al* showed that MSC‐derived exosomes and microparticles can protect osteoarthritis‐derived murine chondrocytes in vitro and in vivo. It was found that exosomes from murine bone marrow–derived MSCs (BM‐MSCs) increased the expression of chondrocyte markers including aggrecan and type II collagen. On the other hand, exosomes inhibited the expression of immune and inflammatory elements responsible for cartilage degradation such as matrix metalloproteinases (MMPs). The exosome‐activated chondrocytes were not able to activate CD4^+^ and CD8^+^ T lymphocytes and B lymphocytes in vitro.^47^ The same results were also observed in the study of Yubao Liu *et al* ^48^ MSC‐derived exosomes were found to inhibit apoptosis in chondrocytes and promote their proliferation. The luciferase activity assay showed that exosomal lncRNA‐KLF3‐AS1 inhibited miR‐206 which in turn facilitates G‐protein‐coupled receptor kinase interacting protein‐1 (GIT1) expression in chondrocytes.^48^ It is shown that GIT1 mediates chondrocyte proliferation and inhibits apoptosis in chondrocytes. The expression of GIT1 is suppressed by miR‐206.^49^, ^50^ In fact, exosomes transfer bioactive molecules including miRNAs and growth factors that can affect many cellular processes such as proliferation and differentiation.^51^ Exosomes derived from MSCs and chondrocytes contain molecules that direct the chondrogenic differentiation of stem cells/progenitors or promote proliferation and migration of chondrocytes. Therefore, they can be beneficial for in vivo treatment of diseases such as OA that is defined by cartilage degradation.^52^

**TABLE 1:** The in vitro chondrogenic potential of exosomes and exosomal miRNAs

Approach	Examples
Extracellular vesicle as an inducer	MSC‐derived exosomes promoted TSPC proliferation and differentiation.^46^ MSC‐derived exosomes re‐induced OA‐like murine chondrocytes markers and inhibit catabolic and inflammatory markers.^47^ Exosomes from human MSCs enhanced proliferation and decreased apoptosis of chondrocytes by increasing the expression of GIT1.^48^ Chondrocyte‐derived and BMSC‐derived exosomes increased chondrogenic markers on CPCs co‐cultured with HUVEC.^72^
Modified extracellular vesicle as an inducer	MiR‐381‐abundant sEVs derived from KGN‐preconditioned hUCMSCs promoted chondrogenesis of hUCMSCs by targeting TAOK1.^55^ Exosomes from miR‐92a‐3p‐overexpressing MSCs increased chondrocyte migration and proliferation by suppressing WNT5A.^57^ Exosomes from miR‐95‐5p‐overexpressing chondrocytes promoted chondrogenic differentiation of MSCs and induced cartilage matrix expression in chondrocytes by inhibition of the expression of HDAC2/8.^58^ Exosomes from miR‐320c‐overexpressing hBMSCs promoted chondrocyte proliferation and migration and enhanced hBMSC chondrogenic differentiation.^61^
Exosomal miRNAs	Exosomal miR‐8485 derived from human chondrocytes enhanced chondrogenic differentiation of BM‐MSCs by regulation of Wnt/β‐catenin pathways.^64^
Extracellular vesicles as a vehicle	Blood‐circulating exosomes were used as a carrier for miR‐140 and enhanced the chondrogenic differentiation of BM‐MSCs in vitro.^65^

Abbreviations

miR: microRNA, BM‐MSCs: bone marrow‐derived mesenchymal stem cells, hUCMSCs: human umbilical cord mesenchymal stem cells, KGN: Kartogenin, TAOK1: TAO kinase 1, sEV: small extracellular vesicles, OA: osteoarthritis, CPC: cartilage progenitor cell, HUVEC: human umbilical vein endothelial cell, MMP: matrix metalloproteinase, ADAMTS5: A disintegrin and metalloproteinase with thrombospondin motifs 5, iNOS: inducible nitric oxide synthase, GIT1: G‐protein‐coupled receptor kinase interacting protein‐1, TSPCs: tendon stem/progenitor cells, WNT5A: Wnt family member 5A and HDAC2/8: histone deacetylase 2/8.

It is well known that exosomal miRNAs mediate many functions of exosomes such as cell proliferation and differentiation as well as inhibition of cell apoptosis.^53^, ^54^ Hui Jing *et al* ^55^ showed that miR‐381‐enrich small extracellular vesicle (sEV) promotes stem cell chondrogenesis in vitro. They obtained miR‐381‐enrich sEV by culturing human umbilical cord mesenchymal stem cells (hUCMSCs) in a medium containing Kartogenin (KGN). KGN is a small drug‐like molecule that promotes chondrogenic differentiation in stem cells and progenitors. This study showed that miR‐381‐3p directly suppresses TAOK1 (TAO Kinase 1) which in turn suppresses Hippo signalling pathway.^55^ Hippo pathway is involved in the promotion of cell apoptosis and inhibition of cell proliferation.^56^ Mao et.al reported that exosomes from miR‐92a‐3p‐overexpressing MSCs increase chondrocyte migration and proliferation. It was found that exosomal miR‐92a‐3p suppresses WNT5A (Wnt family member 5A) that is a key factor in the pathogenesis of OA.^57^ Further study revealed that the exosomes isolated from miR‐95‐5p‐overexpressing chondrocytes promote chondrogenic differentiation of MSCs and induce cartilage matrix expression in chondrocytes. miR‐95‐5p inhibits the expression of histone deacetylase 2/8 (HDAC2/8) that is increased in OA.^58^ It is shown that HDAC2/8, HDAC1 and HDAC3 inhibit the expression of COL2A1 (collagen type II alpha 1 chain) and aggrecan.^59^, ^60^

Hao Sun *et al* evaluated the expression pattern of exosomal microRNAs during chondrogenesis of human bone marrow stem cells (hBMSCs). They found that 35 miRNAs are up‐regulated during chondrogenesis including miR‐320c. Subsequently, they transfected hBMSCs with miR‐320c and found that hBMSC‐320c‐Exos are more powerful in chondrocyte proliferation and chondrogenic differentiation of hBMSCs.^61^ miR‐320c has been found to inhibit the expression of MMP‐13 and Runt‐related transcription factor 2 (RUNX2) that mediate inflammatory responses during cartilage degradation.^62^, ^63^

Chondrocyte‐derived exosomal miR‐8485 was also found to induce BM‐MSC differentiation into chondrocytes. miR‐8485 targets GSK3B (glycogen synthase kinase 3 beta) that finally activates Wnt/β‐catenin pathway and promotes stem cell differentiation into chondrocytes.^64^

In the other study, Gi Won Lee *et al* used blood‐circulating exosomes to transfer miR‐140 into rabbit BM‐MSCs. They showed that exosomes themselves or in a combination with miR‐140 promote chondrogenic differentiation of rabbit BM‐MSCs. Of note, miR‐140 increased the chondrogenic inductivity of exosomes.^65^ This finding can be supported by the other studies that show miR‐140 is involved in cartilage homeostasis and regeneration.^66^, ^67^

## 4 THE IN VITRO THERAPEUTICS OF EVS

MSCs have been known to promote cartilage repair and chondrocyte differentiation through a paracrine effect via cytokine secretion. These factors including transforming growth factor beta (TGF‐β) and hepatocyte growth factor (HGF) constitute a major part of MSCs secretome.^68^ Furthermore, MSCs have been found to secrete chemokines and vascular endothelial growth factor (VEGF) into the synovial fluid to promote cartilage repair in OA patients.^69^, ^70^ It is shown that the secretome of MSCs has a therapeutic function in the treatment of liver, kidney, skin and other organ injuries.^71^ Preclinical studies have shown that exosomes derived from MSCs have promising results in cartilage repair. Yet, the exact mechanisms of tissue repair have not been elucidated. However, it seems that MSCs have a pivotal role in the maintenance of the mesenchymal tissue microenvironment.^11^ Here, the therapeutic functions of EVs derived from MSCs and other sources in the treatment of cartilage repair are discussed. Table 2 summarizes the in vivo functions of EVs in the treatment of cartilage repair.

**TABLE 2:** The in vivo functions of EVs in the treatment of cartilage repair

Function	Cell sources	Animal model	Description
Inducing chondrocyte differentiation	Rat BMSC	Rat patellar tendon defect model	Controlled release of BMSC‐Exos at rat tendons induced the proliferation, migration and differentiation of endogenous tendon stem/progenitor cells (TSPCs).^46^
	Rabbit chondrocytes (CC‐Exos) or BMSCs (BMSC‐Exos)	Mouse model of tendon defect	The increase in CPC proliferation, differentiation and migration, and the ectopic neo‐cartilage formation have been shown in the animal model.
Inducing chondrocyte proliferation	Human MSCs	Collagenase‐induced rat model of OA	Exosomes derived from MSCs were capable to induce chondrocyte proliferation and inhibit cell apoptosis.^76^
	miR‐140‐5p‐overexpressing Synovial mesenchymal stem cells (SMSCs)	OA model of rat Sprague‐Dawley rats	Exosomes derived from miR‐140‐5p‐overexpressing MSCs induced articular chondrocyte (AC) proliferation.^77^
	Human embryonic stem cell–derived MSCs	Rat osteochondral defect model	Exosomes induced chondrocyte proliferation through CD73‐Akt/Erk pathway.^82^
Increasing bioenergetics	Mouse chondrocytes	Mouse model of tendon defect	Exosomes derived from chondrocytes increased the intracellular ATP level in chondrocytes.^87^
Reducing inflammation and immune responses	Human embryonic stem cell–derived MSCs	immunocompetent rat model	Exosomes derived from MSCs induced the infiltration of M2 but not M1 macrophages into the synovial fluid in an immunocompetent rat model and therefore reduce the inflammation.^82^
	Human amniotic fluid stem cell (AFSC)	Rat model of OA	Exosomes directed the polarization of macrophages into M2 type.^106^

### 4.1 Inducing chondrocyte differentiation

Besides the in vitro chondrogenesis role of EVs, some studies have tried to evaluate their potential in the induction of chondrocyte differentiation in animal models. Chen *et al* implanted rabbit CPC (cartilage progenitor cell)‐alginate subcutaneously in mice. Thereafter, exosomes derived from chondrocytes (CC‐Exos) or BMSCs (BMSC‐Exos) were found to be transplanted at the site of implantation. Twelve weeks following implantation, stable cartilage tissue with a high amount of collagen deposition and low vascular ingrowth was observed.^72^ This study showed the potential of exosomes in chondrogenesis and the formation of cartilage tissue. In this study, BMSC‐Exos was used as positive control and the results showed that the CPC proliferation, differentiation and migration are higher in mice treated with BMSC‐Exos when compared to those treated with CC‐Exos. However, there is evidence for ectopic cartilage hypertrophy in animal models treated with BMSC‐Exos and this study showed that CC‐Exos may be more favourable for neo‐cartilage formation. Of note, BMSC or chondrocyte‐derived exosomes exhibit no inflammation and immune responses following exosome transplantation, underlying the advantages of cell‐free secretome therapy in comparison to stem cell therapy.^72^ In the study by Jing *et al*, KGN‐preconditioned small EVs induced transplanted hUCMSCs to differentiate into functional chondrocytes in rabbits with full‐thickness cartilage defects. This induction was attributed to the overexpression of miR‐381‐3p in KGN‐sEVs.^55^

**FIGURE 1:**
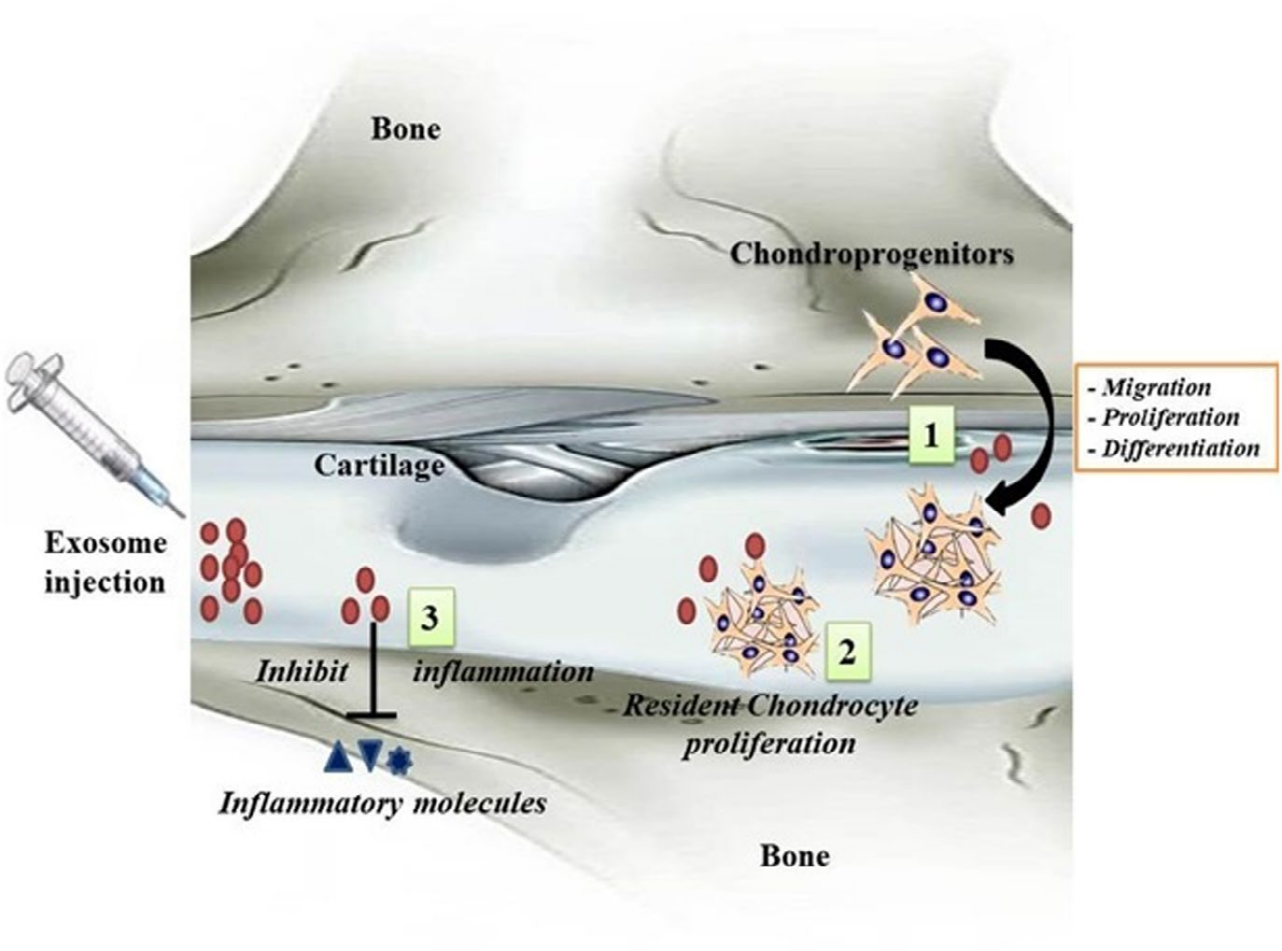
The in vivo chondrogenesis of exosomes. (1) Exosomes are capable to induce transplanted as well as endogenous stem/progenitor cells to migrate, proliferate and differentiate into fully functional chondrocytes. In addition, the stem/progenitor cells are activated to inhibit apoptosis, hypertrophy, fibrosis and inflammation. (2) Exosomes in the cartilage induce the resident chondrocytes to proliferate to maintain and sustain the cartilage. (3) The injected exosomes reduce the inflammation in the damaged cartilage possibly by inhibiting inflammatory molecules such as IL‐1, IL‐6 and TNF‐α, which are responsible for cartilage inflammatory diseases such as OA

Yu *et al* showed that the controlled release of BMSC‐Exos at rat tendons induces the proliferation, migration and differentiation of endogenous tendon stem/progenitor cells (TSPCs) in rat patellar tendon defect model. Following the exosomes‐fibrin injection, a neo‐tendon with a high expression of mohawk, tenomodulin and type I collagen was formed at the site of the defect.^46^ Liu *et al* also showed that the human MSC‐derived exosomes induce the proliferation of chondrocytes and inhibit their apoptosis in an OA rat model. They showed that non‐coding RNAs including lncRNAs and miRNAs are involved in this phenomenon.^48^ Mao *et al* reported that miR‐92a‐3p‐overexpressing exosomes enhance in vivo chondrogenesis and inhibit cartilage degradation by targeting WNT5A in collagenase‐induced mouse model of OA.^57^ The protective effects of exosomes were also observed in the study of Cosenza *et al* ^47^, ^73^ Altogether, these findings indicate that exosomes derived from stem cells or chondrocytes can induce the migration, proliferation and differentiation of transplanted or endogenous stem/progenitor cells, while they increase the proliferation of chondrocytes at the site of tendon defects (Figure 1), suggesting the potential of exosomes in the treatment of cartilage defects in a cell‐free strategy.

### 4.2 Inducing chondrocyte proliferation

The inflammation and oxidative stress in OA cause ECM and cell destruction and loss. Thus, an increase in cell numbers and subsequently an increase in ECM biosynthesis can reduce the OA‐related complications.^74^ The elevated level of proliferative cell nuclear antigen (PCNA), a proliferation marker, has been observed by Zhang *et al* following MSCs‐exosome injection, suggesting the role of exosomes in increasing chondrocytes proliferation in an immunocompetent rat osteochondral defect model. Notably, no inflammation and destructive immune reactions were observed in the rats treated with human MSC‐derived exosomes, showing the importance of cell‐free therapy using EVs.^75^ Further, Liu *et al* reported that the exosomes derived from human MSCs are capable to induce chondrocyte proliferation and inhibit cell apoptosis in the collagenase‐induced rat model of OA.^76^, ^77^

Mechanistically, exosomes have been found to mediate cell proliferation through Akt and Erk1/2 signalling pathways,^78^ and CD73 has a major role in the induction of these pathways.^79^ Damaged tissues release ATP in response to injury or trauma. This extracellular ATP is found to play as a danger signal that activates immune cells to remove the damaged and dead cells. This process also affects the healthy neighbouring cells in a bystander effect. Extracellular ATP and ADP have a short half‐life and are rapidly hydrolysed into AMP.^80^ CD73 from exosomes was found to hydrolyse AMP into adenosine that is a potent activator of survival kinases. These kinases act through kinase receptors involved in the Akt and Erk1/2 signalling pathways, thereby inducing cell proliferation.^81^ Zhang *et al* confirmed the role of the CD73‐Akt/Erk pathway in exosome‐mediated cell proliferation. They showed that the blocking of this pathway by a CD73 inhibitor (AMPCP) and theophylline (an antagonist of adenosine receptor) decreases the number of chondrocytes; however, the matrix synthesis remains unchanged.^82^ Qi *et al* also showed that exosomes derived from MSCs inhibit chondrocyte apoptosis via p38, ERK and Akt pathways, thereby increasing the chondrocyte frequency.^83^ Exosomes from other cell sources have also been shown to induce chondrocyte proliferation. It was reported that exosomes derived from platelet‐rich plasma promote chondrocyte proliferation through the Wnt/β‐catenin signalling pathway.^84^ In contrast, exosomes derived from osteoarthritic chondrocytes inhibited the proliferation and induced the apoptosis of chondrocytes.^85^ Moreover, exosomes isolated from human synovial MSCs (SMSCs) and synovial membrane‐derived MSCs (SMMSCs) were found to induce chondrocyte proliferation and migration, and promote ECM secretion in the cartilage of defected animal model.^77^, ^86^

### 4.3 Increasing bioenergetics

The destruction and dysfunction of mitochondria decrease cell bioenergetics and play a major role in the pathogenesis of OA. It is shown that chondrocytes from OA patients have a lower level of bioenergetics due to reduced mitochondrial biogenesis and decreased mitochondrial electron transport chain (ETC) proteins. A study by Zheng and coworkers showed that exosomes from primary chondrocytes cultured in normal culture contain more mitochondrial proteins than those derived from OA inflammatory environments.^87^ A decrease in mitochondrial proteins leads to the reduction of ATP production, generation of oxidative stress and a perturbation in ECM synthesis by chondrocytes in the cartilage of OA patients. This situation results in inflammation, matrix calcification and catabolism, defective chondrocyte matrix biosynthesis, and cell apoptosis that are the main characteristics of OA.^88^, ^89^ In such a situation, restoring mitochondrial biogenesis and increasing bioenergetics could be helpful in reducing inflammation and inducing regeneration. Exosomes contain a variety of glycolytic enzymes (such as phosphoglucokinase, pyruvate kinase and adenylate kinase) involved in ATP synthesis; they can increase ATP and energy level of resident chondrocytes in vivo.^90^ Therefore, the use of exosomes may alleviate the symptoms of OA through the increase in ATP synthesis. This ATP production is mediated by glycolytic enzymes. Although the ATP production by these enzymes is inefficient compared to the mitochondrial ETC, it is compensated through the increase in the glycolytic flux by a factor of 10‐100.^39^ The glycolysis intermediates are also involved in tissue repair by increasing redox potential.^91^ Although Zheng *et al* showed an elevated intracellular ATP level in exosome‐treated chondrocytes,^87^ there is a lack of information about the role of exosomes from different sources on chondrocyte bioenergetics in both preclinical animal models and the human population.

### 4.4 Reducing inflammation and immune responses

Inflammation is known to be involved in the initiation and development of OA disease.^92^ Following cartilage injury, an inflammatory reaction is triggered through the release of pro‐inflammatory factors such as IL‐1, IL‐8 and MMPs secreted from immune cells, leading to additional ECM destruction in the cartilage of the patients.^93^ These pro‐inflammatory cytokines are through immune cells such as macrophages. Notably, M1 and M2 macrophages are located in the cartilage. Such that CD163^+^ regenerative M2 macrophages support the chondrocyte functions, while CD86^+^ M1 macrophages produce a high amount of pro‐inflammatory cytokines including IL‐1β and TNF‐α.^94^

Since inflammation plays a major role in the pathogenesis of OA, inhibiting inflammatory responses could be helpful in cartilage repair.^95^, ^96^ It is shown that the exosomes derived from MSCs protect the cartilage tissue from the inflammatory responses and ECM component loss. An immunomodulatory function is one of the main MSCs characteristics. This is achieved through the paracrine effects of MSCs by the secretion of trophic factors such as TGF‐β, INF‐γ and HGF.^97^, ^98^, ^99^ Of note, exosomes contain more than 200 immunomodulatory proteins.^100^, ^101^ It has been found that exosome contents increase the level of anti‐inflammatory factors including IL‐10, TGF‐β and INF‐γ and decrease the level of inflammatory factors such as TNF‐α and IL‐1β. In addition, exosomes induce Tregs which in turn suppress the inflammatory responses in OA..^102^ AMSC‐ and BMSC‐derived exosomes have been shown promising results in reducing the inflammation in OA animal models. These sources of MSCs can inhibit the activation of macrophages, weaken the nitric oxide and MMP13 production, and decrease the pro‐inflammatory cytokine production.^47^, ^82^, ^103^, ^104^, ^105^ Furthermore, exosomes from other sources of MSCs have been also shown to suppress cartilage inflammation in animal models. Zhang *et al* showed that exosomes derived from human embryonic stem cell–derived MSCs induce more infiltration of M2 macrophages into the synovial fluid in an immunocompetent rat model.^82^ Zavatti *et al* also reported that exosomes derived from amniotic fluid stem cells (AFSC) change the polarization of macrophages into M2 type. They showed that either MSCs or their exosomes reduce the inflammatory cytokines in an animal model.^106^ It was also reported that exosomes derived from stem cells from human exfoliated deciduous teeth (SHEDs) inhibited cartilage inflammation by inhibiting mTOR pathway which is mediated by miR‐100‐5p.^107^ Exosomes from other cell sources also could inhibit inflammation and suppress immune reactions in cartilage.^84^, ^87^, ^105^, ^107^, ^108^ However, exosomes derived from osteoarthritic chondrocytes have been shown to induce inflammation and increase IL‐1 production by macrophages.^108^

## 5 EXPRESSION PATTERN OF EXOSOMAL MIRNAS DURING CHONDROGENESIS

Exosomal miRNAs have been found to induce the proliferation and differentiation of stem/progenitor cells into chondrocytes.^55^, ^58^ Therefore, it seems that miRNAs are involved in the chondrogenesis function of exosomes. Chondrocytes and MSCs are the main cell sources of these exosomes. MSCs are capable to differentiate into chondrocytes in proper conditions. Defining the exosomal miRNAs preserved or overexpressed during chondrogenic differentiation may help the researchers to explore the miRNA targets involved in the chondrogenic differentiation. Sun *et al* tried to define the expression pattern of exosomal miRNAs during chondrogenesis of BM‐MSCs. They analysed and compared the exosomal miRNAs in BM‐MSCs before and after chondrogenic induction. The microarray data showed that more than 140 miRNAs differentially express by over a twofold change. It was indicated that the expression levels of miR‐1246, miR‐1290, miR‐193a‐5p, miR‐320c and miR‐92a are highly up‐regulated, while the expression levels of miR‐377‐3p and miR‐6891‐5p are dramatically down‐regulated. They also found that the exosomes derived from miR‐320c‐overexpressed MSCs are more potent than normal MSC exosomes in chondrocyte proliferation and matrix deposition.^61^ miR‐320c is known to inhibit MMP‐13 expression and repress the inflammation induced by interleukin‐1β.^109^ These findings show the importance of exosomal miRNAs during chondrogenesis and can help to increase the efficiency of chondrogenesis of exosomes in further studies. However, more in vitro and in vivo studies are required to well define the differentially expressed exosomal miRNAs and their roles in chondrogenesis and cartilage repair. It is also important to compare the chondrogenic potential of exosomal and non‐exosomal miRNAs derived from MSCs and chondrocytes.

## 6 CONCLUSIONS

Many studies indicated the potential role of exosomes in chondrogenesis and cartilage regeneration. Exosomes derived from both chondrocytes and MSCs have been found to induce the differentiation of progenitors and stem cells into mature chondrocytes in vitro. These exosomes have also a capability in the induction of cartilage endogenous stem/progenitor cells to migrate, proliferate and differentiate into chondrocytes in vivo. Exosomes derived from MSCs increase the chondrocyte proliferation and bioenergetics level in the damaged cartilage. They also increase the level of anti‐inflammatory and immunoregulatory cytokines and molecules, and decrease the level of inflammatory cytokines. Therefore, the use of exosomes from MSCs, as a cell‐free therapeutic option, has a great potential in the treatment of OA and other diseases characterized by cartilage damage and chondrocyte loss. However, more studies are needed to evaluate the exact mechanisms of exosomes during cartilage repair. Nevertheless, there are some limitations that should be further considered. The efficient isolation and purification of exosomes are still a challenging task. In some cases, the atrophy of cartilage has been observed following MSC‐derived exosome transplantation. Moreover, sufficient cartilage regeneration is challenging when exosomes from various sources were transplanted.

## CONFLICTS OF INTEREST

We wish to confirm that there are no known conflicts of interest associated with this publication and there has been no significant financial support for this work that could have influenced its outcome.

## Data Availability

Data sharing is not applicable to this article as no new data were created or analysed in this study.
